# Smell and taste recovery in coronavirus disease 2019 patients: a 60-day objective and prospective study

**DOI:** 10.1017/S0022215120001826

**Published:** 2020-08-12

**Authors:** L A Vaira, C Hopkins, M Petrocelli, J R Lechien, C M Chiesa-Estomba, G Salzano, M Cucurullo, F A Salzano, S Saussez, P Boscolo-Rizzo, F Biglioli, G De Riu

**Affiliations:** 1 Maxillofacial Surgery Operative Unit, University Hospital of Sassari, Italy; 2 ENT Department, King's College, London, UK; 3 Maxillofacial Surgery Operative Unit, Bellaria-Maggiore Hospital, Un'azienda Sanitaria Locale (‘AUSL’) Bologna, Italy; 4 COVID-19 Task Force of the Young Otolaryngologists of the International Federation of Oto-rhino-laryngological Societies (‘YO-IFOS’); 5 Department of Human and Experimental Oncology, Faculty of Medicine, UMONS Research Institute for Health Sciences and Technology, University of Mons (‘UMons’), Belgium; 6 Department of Otorhinolaryngology, Osakidetza, Donostia University Hospital, Biodonostia Health Research Institute, San Sebastian, Spain; 7 Maxillofacial Surgery Unit, University Hospital of Naples ‘Federico II’, Italy; 8 Maxillofacial Surgery Department, San Paolo Hospital, Azienda Socio Sanitaria Territoriale (‘ASST’) Santi Paolo e Carlo, University of Milan, Italy; 9 Otolaryngology Operative Unit, Department of Medicine, Surgery and Dentistry, ‘Scuola Medica Salernitana’, University of Salerno, Italy; 10 Department of Neurosciences, Section of Otolaryngology, University of Padua, Treviso, Italy

**Keywords:** COVID-19, Ageusia, Anosmia, Gustatory Dysfunction, SARS-CoV-2, Coronavirus, Hypogeusia, Smell, Taste

## Abstract

**Background:**

The long-term recovery rate of chemosensitive functions in coronavirus disease 2019 patients has not yet been determined.

**Method:**

A multicentre prospective study on 138 coronavirus disease 2019 patients was conducted. Olfactory and gustatory functions were prospectively evaluated for 60 days.

**Results:**

Within the first 4 days of coronavirus disease 2019, 84.8 per cent of patients had chemosensitive dysfunction that gradually improved over the observation period. The most significant increase in chemosensitive scores occurred in the first 10 days for taste and between 10 and 20 days for smell. At the end of the observation period (60 days after symptom onset), 7.2 per cent of the patients still had severe dysfunctions. The risk of developing a long-lasting disorder becomes significant at 10 days for taste (odds ratio = 40.2, 95 per cent confidence interval = 2.204–733.2, *p* = 0.013) and 20 days for smell (odds ratio = 58.5, 95 per cent confidence interval = 3.278–1043.5, *p* = 0.005).

**Conclusion:**

Chemosensitive disturbances persisted in 7.2 per cent of patients 60 days after clinical onset. Specific therapies should be initiated in patients with severe olfactory and gustatory disturbances 20 days after disease onset.

## Introduction

Chemosensitive dysfunction is now considered one of the most frequent symptoms in the early stages of coronavirus disease 2019 (Covid-19).^1–11^ Objective psychophysical evaluation is challenging because of logistical and safety problems. Consequently, as of 1 July 2020, only six retrospective psychophysical studies have been published,^12–17^ and only three of them investigate both olfactory and gustatory functions.^12–14^

In the absence of prospective studies, the long-term recovery rate of chemosensitive function has not yet been determined. Although many authors have reported complete recovery in most patients within a few weeks,^4,5,7,8,10^ psychophysical studies have found that around 25 per cent of patients evaluated 30 days after the clinical onset of Covid-19 have severe chemosensitive disorders (i.e. anosmia, ageusia, severe hyposmia or severe hypogeusia).^12,13^ Clearly, there is still potential for delayed recovery, but the consequence of such a frequent, persistent severe chemosensitive dysfunction, given the high incidence of infection, means that there will be a significant number of patients with potentially long-term morbidity.

In order to understand the longer-term recovery rate of chemosensitive functions, so as to aid the counselling of patients and guide if and when it is appropriate to start a specific therapy, we prospectively evaluated 138 Covid-19 patients with psychophysical tests in 3 Italian hospitals for a period of 60 days from clinical onset of the disease.

## Materials and methods

This multicentre prospective study involved three Italian Covid-19 hospitals: University Hospital of Sassari, San Paolo Hospital in Milan, and Bellaria-Maggiore Hospital in Bologna. The evaluation protocol was approved by an independent ethics committee (approval number: 378-2020-OSS-AUSLBO) and the subjects provided informed consent for participation in the study.

The study included only adult patients aged over 18 years. All patients were symptomatic and presented within 4 days of symptom onset. The patients had a diagnosis of severe acute respiratory syndrome coronavirus-2 (SARS-CoV-2) infection confirmed using reverse transcription polymerase chain reaction analysis of nasopharyngeal swabs. Patients with a history of previous trauma, surgery or radiotherapy in the oral and nasal cavities, allergic rhinitis or rhinosinusitis, or psychiatric or neurological diseases, were excluded from the study.

All patients underwent an evaluation protocol that included psychophysical tests to assess olfactory and gustatory functions every 10 days, for an observation period of 60 days. The first evaluation (baseline) was always performed within 4 days of clinical onset, as per the inclusion criterion. The subsequent assessment (observation time 1) took place 10 days after symptom onset (not baseline). The patient was then evaluated every 10 days for 60 days (observation time 2 to observation time 6).

The evaluation of home-quarantined patients was performed by means of patient self-administered olfactory and gustatory psychophysical tests.^18^ These tests, which have recently been validated for the evaluation of patients in home quarantine using common household odorants and flavours, can be executed remotely by the operator. The evaluation includes an ethyl-alcohol olfactory threshold assessment with nine solutions of decreasing concentration, and a gustatory and olfactory discriminatory function assessment involving seven groups of odorants and four flavoured solutions prepared by the patient.

Hospitalised patients were instead tested with the Connecticut Chemosensory Clinical Research Center orthonasal olfaction test,^19^ a widely used and validated test that includes a butanol threshold evaluation and an odour identification task. A validated discrimination test was carried out to assess taste function, investigating the discriminative capability for four primary tastes.^20^

The evaluation methodology and scoring system of the two olfactory and gustatory psychophysical tests have been previously described in detail.^12,13,18^ The two tests provide standardised olfactory and gustatory scores, on the same severity scale, that can be analysed together.

Statistical analysis was performed with SPSS 26.0 software (IBM, Armonk, New York, USA). Categorical variables are expressed in numerals and percentages of the total. Descriptive statistics for quantitative variables are given as the mean ± standard deviation. The Wilcoxon signed-rank test for paired data was performed to evaluate the statistical significance of changes in olfactory and gustatory scores during the observation period. Logistic regression analysis and Fisher's exact tests were used to evaluate the significance of the correlations between persistent chemosensitive disorders at day 60 and: scores obtained in the different observation times, age, sex, the need for hospitalisation and co-morbidities. The odds ratio was calculated for each variable examined. For the logistic regression analysis, patients were divided into two groups, based on the olfactory and gustatory scores obtained at each observation time: a ‘dysfunction group’ (anosmia or ageusia, or moderate to severe hyposmia or hypogeusia) and a ‘no dysfunction group’ (normal chemosensitive function, mild hyposmia and hypogeusia). The level of statistical significance was set at *p* < 0.05, with a 95 per cent confidence interval.

## Results

A total of 150 Covid-19 patients in the involved hospitals who met the inclusion criteria were enrolled in the study. During the observation period, four patients required invasive ventilation and intensive care unit hospitalisation. In addition, eight patients were lost during follow up for reasons unrelated to health conditions. The data for the 138 patients who completed the evaluation protocol were considered for statistical analysis. General and clinical features of the patients are summarised in Table 1.

**Table 1.** General and clinical characteristics of study population

Characteristic	Values
Gender (*n* (%))	
– Male	68 (49.3)
– Female	70 (50.7)
Age (years)	
– Mean (SD)	51.2 (8.8)
– IQR	46.7–58.0
In-patients (*n* (%))	32 (23.2)
Out-patients (*n* (%))	106 (76.8)
Co-morbidities (*n* (%))	
– Cardiovascular disorder	37 (26.8)
– Pulmonary disorder	21 (15.2)
– Diabetes	15 (10.9)
– BMI >30 kg/m^2^	40 (29)

SD = standard deviation; IQR = interquartile range; BMI = body mass index

**Fig. 1.**
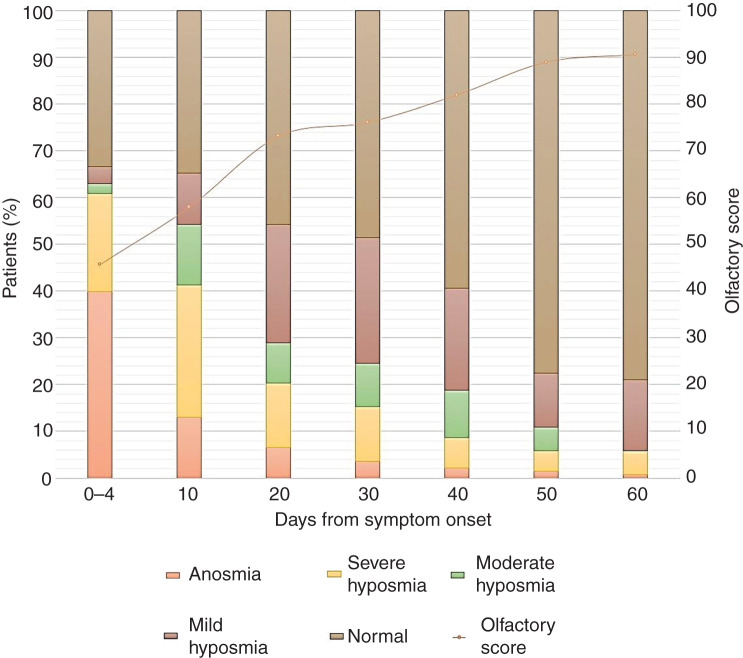
Olfactory clinical diagnosis and trend score during the observation period.

**Fig. 2.**
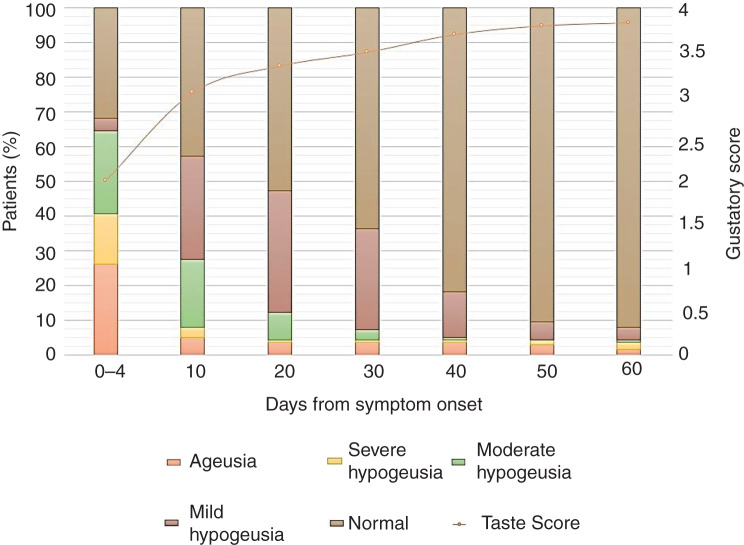
Gustatory clinical diagnosis and trend score during the observation period.

Within the first 4 days of Covid-19 symptom onset, 84.8 per cent of patients had chemosensitive dysfunction (Tables 2 and 3). Specifically, severe olfactory and gustatory disorders (i.e. anosmia or severe hyposmia, and ageusia or severe hypogeusia) affected 60.9 per cent and 40.6 per cent of patients, respectively (Figures 1 and 2). Progressive and statistically significant improvements in olfactory and gustatory scores were observed at every observation point. The most significant increase in gustatory scores occurred within the first 10 days (observation time 1); the most significant improvement in smell took place between 10 (observation time 1) and 20 days (observation time 2) after symptom onset (Tables 2 and 3). At the end of the observation period (observation time 6, 60 days after symptom onset), eight patients (5.8 per cent) still had moderate to severe olfactory dysfunction (Figure 1), while six patients (4.3 per cent) still had a significant taste disorder (Figure 2). Four patients had combined chemosensitive dysfunctions, four patients had isolated smell impairments and two patients had isolated taste disorders.

**Table 2.** Chemosensitive evaluation results: olfactory scores*

Observation time	Olfactory score (median (IQR))	Comparison	Correlation co-efficient	*p*-value
Baseline (T0)	45 (10–90)	T0 *vs* T1	0.920	<0.001
10 days after symptom onset (T1)	50 (40–90)	T1 *vs* T2	0.846	<0.001
20 days after symptom onset (T2)	80 (60–100)	T2 *vs* T3	0.971	<0.001
30 days after symptom onset (T3)	80 (70–100)	T3 *vs* T4	0.957	<0.001
40 days after symptom onset (T4)	90 (70–100)	T4 *vs* T5	0.922	<0.001
50 days after symptom onset (T5)	100 (90–100)	T5 *vs* T6	0.975	<0.001
60 days after symptom onset (T6)	100 (90–100)			

* Of the 138 patients, 22 (15.9 per cent) had isolated taste dysfunction, 69 (50 per cent) had combined dysfunction, 26 (18.8 per cent) had isolated olfactory dysfunction and 21 (15.2 per cent) had normal findings (no chemosensory disorder). IQR = interquartile range

**Table 3.** Chemosensitive evaluation results: gustatory scores*

Observation time	Gustatory score (median (IQR))	Comparison	Correlation co-efficient	*p*-value
Baseline (T0)	2 (0–4)	T0 *vs* T1	0.666	<0.001
10 days after symptom onset (T1)	3 (2–4)	T1 *vs* T2	0.831	<0.001
20 days after symptom onset (T2)	4 (3–4)	T2 *vs* T3	0.919	<0.001
30 days after symptom onset (T3)	4 (3–4)	T3 *vs* T4	0.893	<0.001
40 days after symptom onset (T4)	4 (4–4)	T4 *vs* T5	0.933	<0.001
50 days after symptom onset (T5)	4 (4–4)	T5 *vs* T6	0.975	0.025
60 days after symptom onset (T6)	4 (4–4)			

* Of the 138 patients, 22 (15.9 per cent) had isolated taste dysfunction, 69 (50 per cent) had combined dysfunction, 26 (18.8 per cent) had isolated olfactory dysfunction and 21 (15.2 per cent) had normal findings (no chemosensory disorder). IQR = interquartile range

Any associations between age, gender, need for hospitalisation, cardiovascular and pulmonary co-morbidities, diabetes and obesity and the persistence of chemosensitive disorders at 60 days were assessed with logistic regression analysis (Table 4), but no significant relationships were found.

**Table 4.** Logistic regression and crosstab analysis for anamnestic and clinical features

Variables compared	Normal function at T6 (*n* (%))	Residual dysfunction at T6 (*n* (%))	OR	95% CI for OR		*p*-value
				Lower limit	Upper limit	
IV = age; DV = persistent chemosensitive disorders						
– Age: <50 years	49 (89.1)	6 (10.9)	2.42	0.649	9.003	0.188
– Age: ≥50 years	79 (95.2)	4 (4.8)				
IV = gender; DV = persistent chemosensitive disorders						
– Gender: male	64 (89.2)	4 (10.8)	0.667	0.179	2.475	0.545
– Gender: female	64 (100)	6 (0)				
IV = hospitalisation; DV = persistent chemosensitive disorders						
– Hospitalisation: in-patients	29 (90.6)	3 (9.4)	1.463	0.357	6.109	0.598
– Hospitalisation: out-patients	99 (93.4)	7 (6.6)				
IV = co-morbidities; DV = persistent chemosensitive disorders						
– Co-morbidity: cardiovascular disorder			1.184	0.289	4.845	0.813
– Co-morbidity: pulmonary disorder			0.600	0.072	5	0.637
– Co-morbidity: diabetes			2.211	0.424	11.54	0.364
– Co-morbidity: BMI >30 kg/m^2^			2.657	0.725	9.741	0.140

T6 = observation time 6 (60 days after symptom onset, at the end of the observation period); OR = odds ratio; CI = confidence interval; IV = independent variable; DV = dependant variable

All patients with persistent chemosensitive disorders had severe dysfunction from the beginning of the observation period. However, at baseline (within 4 days of clinical onset), the risk was increased but not significant for both smell and taste. Following the different recovery curves (Figures 1 and 2), the risk of developing a long-lasting severe olfactory disorder, based on the presence of severe dysfunction at the time of observation, became significant at observation time 2 (20 days after symptom onset) (Table 5). As for taste, this risk was significant earlier, at observation time 1 (10 days after symptom onset) (Table 6).

**Table 5.** Olfactory logistic regression and crosstab analysis results

Observation period	Normal function at T6 (*n* (%))	Residual dysfunction at T6 (*n* (%))	OR	95% CI for OR		*p*-value
				Lower limit	Upper limit	
Baseline (T0)						
– Dysfunction group	78 (90.7)	8 (9.3)	11.36	0.642	201.1	0.10
– No dysfunction group	52 (100)	0 (0)				
10 days after symptom onset (T1)						
– Dysfunction group	66 (89.2)	8 (10.8)	16.48	0.932	291.6	0.056
– No dysfunction group	64 (100)	0 (0)				
20 days after symptom onset (T2)						
– Dysfunction group	29 (78.4)	8 (21.6)	58.49	3.278	1043.5	0.005
– No dysfunction group	101 (100)	0 (0)				
30 days after symptom onset (T3)						
– Dysfunction group	26 (76.5)	8 (23.5)	67.03	3.748	1198.8	0.004
– No dysfunction group	104 (100)	0 (0)				
40 days after symptom onset (T4)						
– Dysfunction group	15 (65.2)	8 (34.8)	126.6	6.963	2304.7	0.001
– No dysfunction group	115 (100)	0 (0)				
50 days after symptom onset (T5)						
– Dysfunction group	7 (46.7)	8 (53.3)	279.9	14.71	5326.6	<0.001
– No dysfunction group	123 (100)	0 (0)				

Odds ratios quantify the likelihood of presenting with a residual olfactory dysfunction at the observation time considered. OR = odds ratio; CI = confidence interval

**Table 6.** Gustatory logistic regression and crosstab analysis results

Observation period	Normal function at T6 (*n* (%))	Residual dysfunction at T6 (*n* (%))	OR	95% CI for OR		*p*-value
				Lower limit	Upper limit	
Baseline (T0)						
– Dysfunction group	83 (93.3)	6 (6.7)	7.706	0.425	139.7	0.167
– No dysfunction group	49 (100)	0 (0)				
10 days after symptom onset (T1)						
– Dysfunction group	32 (84.2)	6 (15.8)	40.2	2.204	733.2	0.013
– No dysfunction group	100 (100)	0 (0)				
20 days after symptom onset (T2)						
– Dysfunction group	11 (64.7)	6 (35.3)	137.3	7.266	2596.4	0.001
– No dysfunction group	121 (100)	0 (0)				
30 days after symptom onset (T3)						
– Dysfunction group	4 (40)	6 (60)	371.2	18.0	7645	<0.001
– No dysfunction group	128 (100)	0 (80)				
40 days after symptom onset (T4)						
– Dysfunction group	1 (14.3)	6 (85.7)	1139.6	42.197	30780	<0.001
– No dysfunction group	131 (100)	0 (0)				
50 days after symptom onset (T5)						
– Dysfunction group	0 (0)	6 (100)	3445.0	63.20	187771	<0.001
– No dysfunction group	132 (100)	0 (0)				

Odds ratios quantify the likelihood of presenting with a residual gustatory dysfunction at the observation time considered. OR = odds ratio; CI = confidence interval

## Discussion

This objective and prospective study represents the first psychophysical evaluation of patients in the very early stages of Covid-19. We found no cases of deterioration in terms of the olfactory and gustatory scores from baseline (within 4 days of clinical onset) to observation time 1 (10 days after symptom onset). This finding confirms that chemosensitive disorders are most prevalent at the earliest stages of the disease.

Of the patients, 84.8 per cent had at least one chemosensitive disorder at baseline; this frequency is higher than that reported in some other psychophysical studies which evaluated patients at later stages of the disease.^12–17^ At the end of the observation period, patients presented with mild hyposmia or hypogeusia in 15.2 per cent and 3.6 per cent of cases, respectively. These frequencies are in line with those normally found in the healthy population.^21^ All these cases of mild dysfunction had a more severe disorder at baseline. This finding corroborates the reliability of the prevalence of chemosensitive disturbance at baseline. This parameter could be overestimated if the psychophysical tests were performed only in the later stages of disease, because of potential bias introduced by including patients unaware of having previous mild hyposmia or hypogeusia. Of course, in the absence of testing prior to development of Covid-19, we cannot be sure that the mild hyposmia does represent incomplete recovery, but it is likely that at least some cases reflect pre-existing dysfunction.

• Olfactory and gustatory disturbances in coronavirus disease 2019 patients are frequent and common in the early stages
• In most cases, these symptoms regress completely within 30 days
• Moderate to severe olfactory or gustatory disturbances persisted in 7.2 per cent of patients 60 days after clinical onset
• Specific therapy should be initiated for moderate to severe olfactory and gustatory disturbances persisting 15 days after disease onset, to avoid long-term morbidity

We concur with the findings of Moein *et al*.,^15^ which suggest that self-reported olfactory or gustatory loss underestimates the frequency of chemosensitive disorders. In our series, at baseline, 10.3 per cent of patients who were found to have a chemosensitive disorder on objective testing had self-reported normal function. In the same way, interview-based studies are not able to fully evaluate functional recovery. A significant number of patients with severe chemosensitive dysfunction at baseline self-reported complete recovery, even though mild or moderate hyposmia or hypogeusia persisted according to the objective tests.

Generally, gustatory function has a rapid recovery. Compared to baseline, the number of patients with moderate to severe dysfunction is reduced by 60.7 per cent at 10 days (observation time 1) and is reduced by 80.9 per cent at 20 days (observation time 2) after the clinical onset of disease (Figure 2). Moderate to severe gustatory disorder was still present in six patients (4.3 per cent) at the end of the observation period (observation time 6, 60 days after symptom onset). The recovery of olfactory function was slower, but most notable between observation time 1 and observation time 2 (Table 2). The number of patients with moderate to severe dysfunction at baseline decreased by 13.8 per cent at observation time 1 and decreased by 54 per cent at observation time 2. One month after the onset of symptoms, 39.1 per cent of these patients continued to present with severe or moderate olfactory dysfunction (Figure 1). At the end of the observation period, eight patients (5.8 per cent) presented with residual moderate to severe olfactory disturbance.

The continued improvement of chemosensory function during the whole observation period suggests a pathogenetic mechanism linked to interference of the virus on the taste and smell receptors or to local inflammatory phenomena, rather than central nervous system invasion and permanent neuronal damage.^22–25^ We did not find any predictive factors for the persistence of chemosensitive disorders (Table 3), but our sample may be too small. Larger studies will be needed to determine whether there are predisposing factors for developing long-lasting severe chemosensitive disorders.

Only 10 patients (7.2 per cent) still had severe olfactory or gustatory dysfunction 60 days after symptom onset, which is encouraging news for patients in the early stages of Covid-19, as the majority are likely to recover. However, given the high prevalence of infection in the general population, Covid-19 will still result in a large number of patients with long-term morbidity.

Regarding treatment initiation, it is important to consider both the likelihood of spontaneous recovery and the potential risks of treatments. Our data suggest that the greatest rate of recovery for olfactory disorders occurs between 10 and 20 days, by which time only 20 per cent of patients have severe persisting loss. All patients with moderate hyposmia at 20 days improved further by 60 days. Thus, specific therapy for severe olfactory disorders should be considered if persistent for more than 20 days (observation time 2: odds ratio = 58.5, CI = 3.278–1043.5, *p* = 0.005) (Table 4).

There is a paucity of high-quality evidence regarding potential treatments for post-viral morbidity, and no studies have exclusively investigated olfactory loss following Covid-19 infection. The majority of trials include very small numbers, most lack blinding, randomisation and a control arm, and rates of improvement are usually no greater than reported rates of spontaneous improvement. It is also difficult to compare the current cohort of patients to those seen previously with post-viral loss, as the focus on anosmia during the pandemic has allowed us the unique opportunity to study patients with post-Covid-19 loss at a much earlier stage. However, we can make some recommendations on treatment based on previous studies.

Studies of olfactory training suggest small to moderate benefits in terms of both identification and discrimination, but not thresholds compared to control groups.^26^ Although the effect size may be small, all patients with severe loss should be encouraged to undertake olfactory training, as the risk of harm is minimal.

Oral steroids, but not topical steroids, were shown to improve olfactory function in a group of patients including post-viral anosmia cases.^27^ However, there are concerns regarding the use of systemic steroids in cases with or at risk of severe acute respiratory Covid-19. For instance, a systematic review of usage in influenza cases suggests possible harm.^28^ Furthermore, delayed viral clearance has been previously demonstrated in Middle East respiratory syndrome.^29^ However, if decisions regarding usage are delayed until day 20, the risks of developing long-term morbidity will be significantly reduced.

Alpha-lipoic acid has been shown to improve the results of objective tests of olfactory function in an uncontrolled study,^30^ but its use can be associated with neurological side effects, including headache, dizziness and confusion, which may be difficult to interpret alongside Covid-19 manifestations. Omega-3 supplementation was found to be protective against olfactory loss during the recovery period after skull base surgery and therefore may have potential in aiding recovery after post-viral olfactory loss,^31^ although this has not been formally tested in Covid-19 patients. Intranasal vitamin A added to olfactory training resulted in greater rates of improvement compared with olfactory training alone,^32^ but it is locally irritant to the nose.

Medical treatments specifically aimed at hypogeusia are even more limited and no specific recommendations can be made.

The differences between the chemosensitive scores at observation time 5 (50 days after symptom onset) and observation time 6 (60 days after symptom onset) were significant. Hence, in some cases, the recovery process is still ongoing 60 days after clinical onset of the disease. We will continue to follow these patients to understand whether Covid-19 is capable of causing permanent olfactory or gustatory disturbances. Histological analysis of samples taken from these patients may be useful in determining the pathogenesis of these disorders.

## Conclusion

Olfactory and gustatory disturbances in Covid-19 patients are frequent and common in the early stages of the disease. In most cases, they resolve completely within 30 days. Moderate to severe olfactory or gustatory disturbance persisted in 7.2 per cent of patients 60 days after clinical onset of the disease. In order to avoid long-term morbidity, specific therapies should be initiated in patients with moderate to severe olfactory disturbance 20 days after disease onset.
